# Statistical power as a function of Cronbach alpha of instrument questionnaire items

**DOI:** 10.1186/s12874-015-0070-6

**Published:** 2015-10-14

**Authors:** Moonseong Heo, Namhee Kim, Myles S. Faith

**Affiliations:** Department of Epidemiology and Population Health, Albert Einstein College of Medicine, 1300 Morris Park Avenue, Bronx, NY 10461 USA; Department of Radiology, Albert Einstein College of Medicine, 1300 Morris Park Avenue, Bronx, NY 10461 USA; Department of Nutrition, Gillings School of Public Health, University of North Carolina—Chapel Hill, Chapel Hill, NC 27599 USA

**Keywords:** Cronbach alpha, Coefficient alpha, Test-retest correlation, Internal consistency, Reliability, Statistical power, Effect size

## Abstract

**Background:**

In countless number of clinical trials, measurements of outcomes rely on instrument questionnaire items which however often suffer measurement error problems which in turn affect statistical power of study designs. The Cronbach alpha or coefficient alpha, here denoted by *C*_*α*_, can be used as a measure of internal consistency of parallel instrument items that are developed to measure a target unidimensional outcome construct. Scale score for the target construct is often represented by the sum of the item scores. However, power functions based on *C*_*α*_ have been lacking for various study designs.

**Methods:**

We formulate a statistical model for parallel items to derive power functions as a function of *C*_*α*_ under several study designs. To this end, we assume fixed true score variance assumption as opposed to usual fixed total variance assumption. That assumption is critical and practically relevant to show that smaller measurement errors are inversely associated with higher inter-item correlations, and thus that greater *C*_*α*_ is associated with greater statistical power. We compare the derived theoretical statistical power with empirical power obtained through Monte Carlo simulations for the following comparisons: one-sample comparison of pre- and post-treatment mean differences, two-sample comparison of pre-post mean differences between groups, and two-sample comparison of mean differences between groups.

**Results:**

It is shown that *C*_*α*_ is the same as a test-retest correlation of the scale scores of parallel items, which enables testing significance of *C*_*α*_. Closed-form power functions and samples size determination formulas are derived in terms of *C*_*α*_, for all of the aforementioned comparisons. Power functions are shown to be an increasing function of *C*_*α*_, regardless of comparison of interest. The derived power functions are well validated by simulation studies that show that the magnitudes of theoretical power are virtually identical to those of the empirical power.

**Conclusion:**

Regardless of research designs or settings, in order to increase statistical power, development and use of instruments with greater *C*_*α*_, or equivalently with greater inter-item correlations, is crucial for trials that intend to use questionnaire items for measuring research outcomes.

**Discussion:**

Further development of the power functions for binary or ordinal item scores and under more general item correlation strutures reflecting more real world situations would be a valuable future study.

## Background

Use of instrument questionnaire items is essential for measurement of outcome of interest in innumerable numbers of clinical trials. Many trials use well-established instruments; for example, major depressive disorders are often evaluated by scores on the Hamilton Rating Scale of Depression (HRSD) [1] in psychiatry trials. However, it is by far more often the case when instruments germane to a research outcome are not available. In such cases, of course, questionnaire items need to be developed to measure the outcome, and their psychometric properties should be evaluated for construct validity, internal consistency, and reliability among others [2, 3]. The internal consistency of instrument items quantifies how similarly in a interrelated fashion the items represent an outcome construct that the instrument is aiming to measure [4], whereas reliability is defined as the squared correlation between true score and observed score [3].

Cronbach alpha also known as coefficient alpha [5], hereafter denoted by *C*_*α*_, has been very widely used to quantify the internal consistency and reliability of items in clinical research and beyond [6] although internal consistency and reliability are not exchangeable psychometric concepts in general. For this reason, some argue that *C*_*α*_ should not be used for quantifying either concept (e.g.,[7, 8]). One the other hand, for special cases where items under study are parallel such that items are designed as replicates to measure a unidimensional construct or attribute, *C*_*α*_ can quantify internal consistency and reliability as well [2] although in general *C*_*α*_ is not necessarily a measure of unidimensionality or homogeneity [4, 8]. In this paper, we consider parallel items; for example, items within a same factor could be considered parallel for a unidimensional construct. In this sense, items of HRSD are not parallel since it measures depression, a multidimensional construct with many factors.

The Cronbach alpha by mathematical definition is an adjusted proportion of total variance of the item scores explained by the sum of covariances between item scores, and thus ranges between 0 and 1 if all covariance elements are non-negative. Specifically, for an instrument with *k* items with a general covariance matrix Σ among the item scores, *C*_*α*_ is defined as

1 $${\mathit{\mathsf{C}}}_{\alpha }=\frac{\mathit{\mathsf{k}}}{\mathit{\mathsf{k}}-\mathsf{1}}\left(\frac{{\text{\textbf{\textsf{1}}}}^{\mathit{\mathsf{T}}}\boldsymbol{\Sigma}\text{\textbf{\textsf{1}}}-\mathit{\mathsf{trac}}e\left(\boldsymbol{\Sigma}\right)}{{\text{\textbf{\textsf{1}}}}^{\mathit{\mathsf{T}}}\boldsymbol{\Sigma}\text{\textbf{\textsf{1}}}}\right)=\frac{\mathit{\mathsf{k}}}{\mathit{\mathsf{k}}-\mathsf{1}}\left(\mathsf{1}-\frac{\mathit{\mathsf{trac}}e\left(\boldsymbol{\Sigma}\right)}{{\text{\textbf{\textsf{1}}}}^{\mathit{\mathsf{T}}}\boldsymbol{\Sigma}\text{\textbf{\textsf{1}}}}\right),$$

where *trace*(.) is the sum of the diagonal elements of a square matrix, **1** is a column vector with *k* unit elements, and **1**^*T*^ is the transpose of **1**. This quantification is therefore based on the notion that relative magnitudes of covariances between item scores compared to those of corresponding variances serves as a measure of similarities of the items. Consequently, items with higher *C*_*α*_ are preferred to measure the target outcome. However, *C*_*α*_ is a lower bound for reliability, but is not equal to reliability unless the items are parallel or essentially τ-equivalent [3, 8]. The sum of the instrument items serves as a scale for the outcome, and is used for statistical inference including testing statistical hypotheses. At the design stage of clinical trials, information about magnitude of reliability or internal consistency of developed parallel items is crucial for power analysis and sample size determinations. Nonetheless, power functions based on *C*_*α*_ have been lacking for various study designs.

In this paper, to derive closed-from power functions, we formulate a statistical model for parallel items that relates the item scores to a measurement error problem. Under this model, *C*_*α*_ (1) is explicitly expressed in terms of an inter-item correlation. We examine relationship among *C*_*α*_, a test-retest correlation and reliability of scale scores that enables testing significance of *C*_*α*_ through Fisher z-transformation. We explicitly express statistical power as a function of *C*_*α*_ for the following comparisons: one-sample comparison of pre- and post-treatment mean differences, two-sample comparison of pre-post mean differences between groups, and two-sample comparison of mean differences between groups. Simulation study results compare derived theoretical power with empirical power and discussion and conclusion follow.

## Methods

### Statistical model

We consider the following model for item score *Y*_*ij*_ to the *j*-th parallel item for the *i*-th subject:

2 $${\mathit{\mathsf{Y}}}_{\mathit{\mathsf{i}}\mathit{\mathsf{j}}}={\mu}_{\mathit{\mathsf{i}}}+{e}_{\mathit{\mathsf{i}}\mathit{\mathsf{j}}}$$

The parameter *μ*_*i*_ represents the “true score” of the target (outcome) construct for the *i*-th subject. At the population level, its expectation and variance are assumed to be $\mathit{\mathsf{E}}\left({\mu}_{\mathit{\mathsf{i}}}\right)=\mu$ and $\mathit{\mathsf{V}}\mathit{\mathsf{a}}\mathit{\mathsf{r}}\left({\mu}_{\mathit{\mathsf{i}}}\right)={\sigma}_{\mu}^{\mathsf{2}}$, which we call the *true score variance*. The error term ${e}_{\mathit{\mathsf{i}}\mathit{\mathsf{j}}}$ represents the deviate of the item score *Y*_*ij*_ from the true score *μ*_*i*_, i.e., ${e}_{\mathit{\mathsf{i}}\mathit{\mathsf{j}}}$ is the measurement error of *Y*_*ij*_. The expectation and variance of ${e}_{\mathit{\mathsf{i}}\mathit{\mathsf{j}}}$ for all subjects are assumed to be $\mathit{\mathsf{E}}\left({e}_{\mathit{\mathsf{i}}\mathit{\mathsf{j}}}\right)=\mathsf{0}$, i.e., unbiasedness assumption, that is, *E*_*j*_(*Y*_*ij*_) = *μ*_*i*_ and *E*_*i*_*E*_*j*_(*Y*_*ij*_) = *E*(*μ*_*i*_) = *μ*, where *E*_*j*_ denotes the expectation over *j*. It is also assumed that $\mathit{\mathsf{V}}\mathit{\mathsf{a}}\mathit{\mathsf{r}}\left({e}_{\mathit{\mathsf{i}}\mathit{\mathsf{j}}}\right)={\sigma}_e^{\mathsf{2}}$, which we call the *measurement error variance*. We further assume the following: *μ*_*i*_ and *e*_*ij*_ are mutually independent, i.e., *μ*_*i*_ ⊥ *e*_*ij*_; and the elements of *e*_*ij*_’s are independent for a given subject, i.e., conditional independence, that is, *e*_*ij*_ ⊥ *e*_*ij*′_|*μ*_*i*_ for *j* ≠ *j*′. Note that this conditional independence does not imply marginal impendence between *Y*_*ij*_ and *Y*_*ij*′_. In short, model (2) is a mixed-effects linear model for data with a two-level structure in a way that repeated item scores are nested within individuals.

Under those assumptions, we have $\mathit{\mathsf{V}}\mathit{\mathsf{a}}\mathit{\mathsf{r}}\left({\mathit{\mathsf{Y}}}_{\mathit{\mathsf{i}}\mathit{\mathsf{j}}}\right)\equiv {\sigma}^{\mathsf{2}}={\sigma}_{\mu}^{\mathsf{2}}+{\sigma}_e^{\mathsf{2}}$, that is, the *total variance* of the item scores is the sum of the true score variance and the measurement error variance. Inter-item (score) covariance can be obtained as $\mathit{\mathsf{C}}\mathit{\mathsf{o}}\mathit{\mathsf{v}}\left({\mathit{\mathsf{Y}}}_{\mathit{\mathsf{i}}\mathit{\mathsf{j}}},{\mathit{\mathsf{Y}}}_{\mathit{\mathsf{i}}{\mathit{\mathsf{j}}}^{\prime }}\right)={\sigma}_{\mu}^{\mathsf{2}}$ for *j* ≠ *j*′. Therefore, the diagonal elements of covariance matrix Σ under model (2) are identical and so are the off-diagonal elements. This compound symmetry covariance structure, also known as essential τ-equivalence, is the covariance matrix of parallel items each of which targets the underlying true score for a unidimensional construct. Furthermore, the compound symmetry covariance structure can be regarded as a covariance matrix of “standardized” item scores with unequal variances and covariances. Inter-item (score) correlation, denoted here by *ρ*, can accordingly be obtained as

3 $$\mathit{\mathsf{Corr}}\left({\mathit{\mathsf{Y}}}_{\mathit{\mathsf{i}}\mathit{\mathsf{j}}}{\mathit{\mathsf{Y}}}_{\mathit{\mathsf{i}}{\mathit{\mathsf{j}}}^{\prime }}\right)\equiv \rho =\frac{\sigma_{\mu}^{\mathsf{2}}}{\sigma^{\mathsf{2}}}=\frac{\sigma_{\mu}^{\mathsf{2}}}{\sigma_{\mu}^{\mathsf{2}}+{\sigma}_e^{\mathsf{2}}}.$$

Although item scores are correlated within subjects, they are independent between subjects. Note that this inter-item correlation is not necessarily equal to item-score reliability that quantifies a correlation between true and observed scores.

In this paper, we assume that the true score variance ${\sigma}_{\mu}^{\mathsf{2}}$, instead of the total variance σ^2^, is fixed at the population level, and it does not depend on the item scores of the subjects. Stated differently, the total variance σ^2^ depends only on ${\sigma}_e^{\mathsf{2}}$ which depends on item scores and thus σ^2^ is assumed to be an increasing function of only measurement errors of the item scores. Let us call this assumption the *fixed true score variance assumption*, which is crucial and reasonable from the perspective of measurement error theory in general. This assumption is crucial because it makes the total variance as a function of only measurement error variance as mentioned above, and it is reasonable because at the population level true score variance should not be varying whereas magnitudes of measurement error variance depend on reliability of items. Consequently, the true score variance ${\sigma}_{\mu}^{\mathsf{2}}$ is not a function of inter-item correlation *ρ*, but the measurement error variance ${\sigma}_e^{\mathsf{2}}$ is a decreasing function of *ρ* since from equation (3) we have

4 $${\sigma}_e^{\mathsf{2}}=\left(\mathsf{1}-\rho \right){\sigma}^{\mathsf{2}}=\left(\mathsf{1}/\rho -\mathsf{1}\right){\sigma}_{\mu}^{\mathsf{2}}.$$

It follows that as the item scores are closer or more similar to each other within subjects, the measurement errors will be smaller, which follows that the total variance is also a decreasing function of *ρ* since

5 $${\sigma}^{\mathsf{2}}={\sigma}_{\mu}^{\mathsf{2}}+{\sigma}_e^{\mathsf{2}}={\sigma}_{\mu}^{\mathsf{2}}/\rho .$$

We assume that the magnitudes of both ${\sigma}_e^{\mathsf{2}}$ and ${\sigma}_{\mu}^{\mathsf{2}}$ are known and thus that of σ^2^ for the purpose of derivation of power functions based on normal destructions instead of *t*-distributions, although replacement by *t*-distributions should be straightforward yet with little difference in results for sizable sample sizes.

### Cronbach alpha, scale score and its variance

We assume that there are *k* items in an instrument, i.e., *j* =1, 2, …, *k*. The *C*_*α*_ (1) of *k* items under model (2) and aforementioned assumptions can be expressed as

6 $${\mathit{\mathsf{C}}}_{\alpha }=\frac{\mathit{\mathsf{k}}{\sigma}_{\mu}^{\mathsf{2}}}{\sigma_e^{\mathsf{2}}+\mathit{\mathsf{k}}{\sigma}_{\mu}^{\mathsf{2}}}=\frac{\mathit{\mathsf{k}}\rho }{\mathsf{1}+\rho \left(\mathit{\mathsf{k}}-\mathsf{1}\right)}.$$

**Fig. 1:**
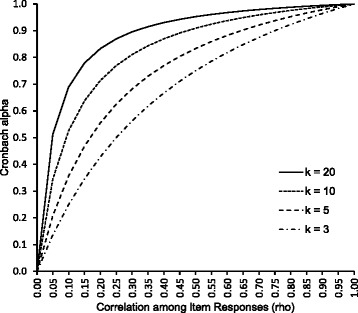
Relationship between Cronbach alpha (*C*_*α*_) and inter-item correlation (*ρ*) over varying number of items (*k*)

It is due to the fact that $\boldsymbol{\Sigma}={\sigma}_e^{\mathsf{2}}\text{\textbf{\textsf{I}}}+{\sigma}_{\mu}^{\mathsf{2}}\text{\textbf{\textsf{1}}}{\text{\textbf{\textsf{1}}}}^{\mathit{\mathsf{T}}}$ under model (2) where **I** is a *k*-by-*k* identity matrix. *C*_*α*_ in equation (6) is seen to be an increasing function of both *ρ* and *k* as depicted in Fig. 1. Therefore, the number of items needs to be fixed for comparison of *C*_*α*_ of several candidate sets of items. It follows that for a fixed number of items, higher *C*_*α*_ is associated with smaller measurement error of items through higher inter-item correlation *ρ*. From equation (6), *ρ* can be expressed in terms of *C*_*α*_ as follows:

7 $$\rho =\frac{{\mathit{\mathsf{C}}}_{\alpha }}{\mathit{\mathsf{k}}-{\mathit{\mathsf{C}}}_{\alpha}\left(\mathit{\mathsf{k}}-\mathsf{1}\right)}.$$

Of note, the corresponding correlation matrix is denoted by $\text{\textbf{\textsf{P}}}=\left(\mathsf{1}-\rho \right)\text{\textbf{\textsf{I}}}+\rho \text{\textbf{\textsf{1}}}{\text{\textbf{\textsf{1}}}}^{\mathit{\mathsf{T}}}$, an equi-correlation matrix.

The *k* correlated items are often summed up to a scale that is intended to measure the target construct. The scale score is denoted here by

$${\mathit{\mathsf{S}}}_{\mathit{\mathsf{i}}}={\displaystyle \sum_{\mathit{\mathsf{j}}=\mathsf{1}}^{\mathit{\mathsf{k}}}{\mathit{\mathsf{Y}}}_{\mathit{\mathsf{i}}\mathit{\mathsf{j}}}},$$

which can be viewed as an observed summary score for the *i*-th subject. Suppressing the subscription *i* in *S*_*i*_, its mean and variance can be obtained as follows:

8 $${E}_j(S)=k{\mu}_i,$$

and

9 $$Var(S)=k{\sigma}^2\left\{1+\rho \left(k-1\right)\right\}.$$

With respect to the mean (8), average scale score *S*_*i*_/*k* when used as observed score is an unbiased estimate of true score *μ*_*i*_ for the *i*-th subject. The reliability, denoted here by *R*, defined as the squared correlation between true score and observed score can be obtained as follows:

10 $$\mathit{\mathsf{R}}=\mathit{\mathsf{C}}\mathit{\mathsf{o}}\mathit{\mathsf{r}}{\mathit{\mathsf{r}}}^{\mathsf{2}}\left({\mathit{\mathsf{S}}}_{\mathit{\mathsf{i}}}/\mathit{\mathsf{k}},{\mu}_{\mathit{\mathsf{i}}}\right)=\frac{\mathit{\mathsf{k}}\rho }{\mathsf{1}+\rho \left(\mathit{\mathsf{k}}-\mathsf{1}\right)}={\mathit{\mathsf{C}}}_{\alpha }.$$

This equation supports Theorem 3.1 of Novick and Lewis [9] that *R* = *C*_*α*_ if and only if the items are parallel. Since statistical analysis results do not depend on whether *S*_*i*_/*k* or *S*_*i*_ is used, we use the sum *S* in what follows.

With respect the total variance (9), if the total variance, instead of the true score variance, is assumed to be fixed, *Var*(S) is an increasing function of *ρ*, which conforms to an elementary statistical theory that variance of sum of correlated variables increases with increasing correlation. On the contrary, under the fixed true score variance assumption, it can be seen that *Var*(S) is a decreasing function of *ρ* since equation (9) can be re-expressed in terms of ${\sigma}_{\mu}^{\mathsf{2}}$ via equation (5) as follows:

11 $$\mathit{\mathsf{V}}\mathit{\mathsf{a}}\mathit{\mathsf{r}}\left(\mathit{\mathsf{S}}\right)=\mathit{\mathsf{k}}{\sigma}_{\mu}^{\mathsf{2}}\left(\mathsf{1}/\rho +\mathit{\mathsf{k}}-\mathsf{1}\right)={\mathit{\mathsf{k}}}^{\mathsf{2}}{\sigma}_{\mu}^{\mathsf{2}}/{\mathit{\mathsf{C}}}_{\alpha }.$$

The last equation is due to equation (7). It follows that *Var*(S) is also a decreasing function of *C*_*α*_. In sum, increase of *ρ* decreases the magnitude of *σ*^2^ which in turn decreases the magnitude of *Var*(*S*); therefore such indirect decreasing effect of *ρ* on *Var*(*S*) is larger than direct increasing effect of *ρ* on *Var*(*S*) in equation (9).

### Cronbach alpha and test-retest correlation

Reliability *R* of instruments is sometimes evaluated by test-retest correlation [3]. Based on model (2), the test and retest item scores can be specified as ${\mathit{\mathsf{Y}}}_{\mathit{\mathsf{i}}\mathit{\mathsf{j}}}^{\mathit{\mathsf{t}}e\mathit{\mathsf{s}}\mathit{\mathsf{t}}}={\mu}_{\mathit{\mathsf{i}}}+{e}_{\mathit{\mathsf{i}}\mathit{\mathsf{j}}}$ and ${\mathit{\mathsf{Y}}}_{\mathit{\mathsf{i}}\mathit{\mathsf{j}}}^{\mathit{\mathsf{r}}e\mathit{\mathsf{t}}e\mathit{\mathsf{s}}\mathit{\mathsf{t}}}={\mu}_{\mathit{\mathsf{i}}}+{e}_{\mathit{\mathsf{i}}\mathit{\mathsf{j}}}$, respectively with a common *μ*_*i*_ for both test and retest scores for each subject, *i* = 1, 2,…, *N*. The test-retest correlation can then be measured by the correlation, denoted by *Corr*(*S*_*test*_, *S*_*retest*_), between scale scores ${\mathit{\mathsf{S}}}_{\mathit{\mathsf{t}}e\mathit{\mathsf{s}}\mathit{\mathsf{t}}}={\displaystyle {\sum}_{\mathit{\mathsf{j}}=\mathsf{1}}^{\mathit{\mathsf{k}}}{\mathit{\mathsf{Y}}}_{\mathit{\mathsf{i}}\mathit{\mathsf{j}}}^{\mathit{\mathsf{t}}e\mathit{\mathsf{s}}\mathit{\mathsf{t}}}}$ and ${\mathit{\mathsf{S}}}_{\mathit{\mathsf{r}}e\mathit{\mathsf{t}}e\mathit{\mathsf{s}}\mathit{\mathsf{t}}}={\displaystyle {\sum}_{\mathit{\mathsf{j}}=\mathsf{1}}^{\mathit{\mathsf{k}}}{\mathit{\mathsf{Y}}}_{\mathit{\mathsf{i}}\mathit{\mathsf{j}}}^{\mathit{\mathsf{r}}e\mathit{\mathsf{t}}e\mathit{\mathsf{s}}\mathit{\mathsf{t}}}}$ representing the scale scores of test and retest, respectively. Under the aforementioned assumptions for model (2) it can be shown that

12 $$Cov\left({S}_{test},\kern0.5em {S}_{retest}\right)={k}^2\rho {\sigma}^2,$$

and from equation (10)

13 $$Var\left({S}_{test}\right)=Var\left({S}_{retest}\right)=k{\sigma}^2\left\{1+\rho \left(k-1\right)\right\}.$$

It follows that:

14 $$\mathit{\mathsf{C}\mathsf{orr}}\left({\mathit{\mathsf{S}}}_{\mathit{\mathsf{t}}e\mathit{\mathsf{s}}\mathit{\mathsf{t}}},{\mathit{\mathsf{S}}}_{\mathit{\mathsf{r}}e\mathit{\mathsf{t}}e\mathit{\mathsf{s}}\mathit{\mathsf{t}}}\right)=\frac{\mathit{\mathsf{C}}\mathit{\mathsf{o}}\mathit{\mathsf{v}}\left({\mathit{\mathsf{S}}}_{\mathit{\mathsf{t}}e\mathit{\mathsf{s}}\mathit{\mathsf{t}}}{\mathit{\mathsf{S}}}_{\mathit{\mathsf{r}}e\mathit{\mathsf{t}}e\mathit{\mathsf{s}}\mathit{\mathsf{t}}}\right)}{\sqrt{\mathit{\mathsf{V}}\mathit{\mathsf{a}}\mathit{\mathsf{r}}\left({\mathit{\mathsf{S}}}_{\mathit{\mathsf{t}}e\mathit{\mathsf{s}}\mathit{\mathsf{t}}}\right)}\sqrt{\mathit{\mathsf{V}}\mathit{\mathsf{a}}\mathit{\mathsf{r}}\left({\mathit{\mathsf{S}}}_{\mathit{\mathsf{t}}e\mathit{\mathsf{s}}\mathit{\mathsf{t}}}\right)}}=\frac{\mathit{\mathsf{k}}\rho }{\mathsf{1}+\rho \left(\mathit{\mathsf{k}}-\mathsf{1}\right)}=\mathit{\mathsf{R}}={\mathit{\mathsf{C}}}_{\alpha }.$$

This equation shows that the test-rest correlation is the same as both *C*_*α*_ and *R* due to equations (6) and (10), which provides another interpretation of *C*_*α*_. This property is especially useful when there is only one item available, in which case estimation of *C*_*α*_ or *ρ* is impossible by definition. However, the test and retest scores can be thought of as two correlated parallel item scores, and thus their correlation can serve as *C*_*α*_ of the single item. It is particularly fitting since *ρ* = *C*_*α*_ = *R* based on either equation (6), (7), or (14) when *k* = 1.

Taken together, the power ${\varphi}_{{\mathit{\mathsf{C}}}_{\alpha }}$ of testing significance of *C*_*α*_ against any null value should be equivalent to that of testing significance of a correlation using a Fisher’s z-transformation as long as items are parallel, that is,

$${\varphi}_{{\mathit{\mathsf{C}}}_{\alpha }}=\mathsf{1}-\varPhi \left[{\varPhi}^{-\mathsf{1}}\left(\mathsf{1}-\alpha /\mathsf{2}\right)-\sqrt{\mathit{\mathsf{N}}-\mathsf{3}}\left(\frac{\mathsf{1}}{\mathsf{2}} \ln \left(\frac{\mathsf{1}+{\mathit{\mathsf{C}}}_{\alpha }}{\mathsf{1}-{\mathit{\mathsf{C}}}_{\alpha }}\right)+\frac{{\mathit{\mathsf{C}}}_{\alpha }}{\mathsf{2}\left(\mathit{\mathsf{N}}-\mathsf{1}\right)}\right)\right]$$

for a two-tailed significance level *α*, where Φ is the cumulative distribution function of a standardized normal distribution, and Φ^−1^ is its inverse function, i.e., Φ(Φ^−1^(*x*)) = Φ^−1^(Φ(*x*)) = *x*. We note that although it is necessary to be added for validation of unbiasedness of the test statistics under the null hypothesis, the probability under the other rejection area will be ignored for all test statistics considered herein. For general covariance structures for non-parallel items, however, many other tests for significance of reliability and *C*_*α*_ have been developed [10–17].

### Pre-post comparison

We consider application of a paired *t*-test to the case of comparison of within-group means of scale scores between pre- and post-interventions. Based on model (2), the pre- and post-intervention item scores can be specified as ${\mathit{\mathsf{Y}}}_{\mathit{\mathsf{i}}\mathit{\mathsf{j}}}^{\mathit{\mathsf{p}}\mathit{\mathsf{r}}e}={\mu}_{\mathit{\mathsf{i}}}+{e}_{\mathit{\mathsf{i}}\mathit{\mathsf{j}}}$ and ${\mathit{\mathsf{Y}}}_{\mathit{\mathsf{i}}\mathit{\mathsf{j}}}^{\mathit{\mathsf{post}}}={\mu}_{\mathit{\mathsf{i}}}+{\delta}_{\mathit{\mathsf{P}}\mathit{\mathsf{P}}}+{e}_{\mathit{\mathsf{i}}\mathit{\mathsf{j}}}$, respectively; the mean of the post-intervention item scores are shifted by *δ*_*PP*_, an intervention effect. Consequently, we have

15 $$E\left({S}_{post}\right)-E\left({S}_{pre}\right)=k{\delta}_{pp},$$

where ${\mathit{\mathsf{S}}}_{\mathit{\mathsf{p}}\mathit{\mathsf{r}}e}={\displaystyle {\sum}_{\mathit{\mathsf{j}}=\mathsf{1}}^{\mathit{\mathsf{k}}}{\mathit{\mathsf{Y}}}_{\mathit{\mathsf{i}}\mathit{\mathsf{j}}}^{\mathit{\mathsf{p}}\mathit{\mathsf{r}}e}}$ and ${\mathit{\mathsf{S}}}_{\mathit{\mathsf{post}}}={\displaystyle {\sum}_{\mathit{\mathsf{j}}=\mathsf{1}}^{\mathit{\mathsf{k}}}{\mathit{\mathsf{Y}}}_{\mathit{\mathsf{i}}\mathit{\mathsf{j}}}^{\mathit{\mathsf{post}}}}$ are the pre- and post-intervention scale scores, respectively. A moment estimate of *δ*_*PP*_ from (15) can be estimated as

16 $${\widehat{\delta}}_{\mathit{\mathsf{P}}\mathit{\mathsf{P}}}=\left({\overline{\mathit{\mathsf{S}}}}_{\mathit{\mathsf{p}\mathsf{ost}}}-{\overline{\mathit{\mathsf{S}}}}_{\mathit{\mathsf{p}}\mathit{\mathsf{r}}e}\right)/\mathit{\mathsf{k}},$$

where $\overline{\mathit{\mathsf{S}}}={\displaystyle {\sum}_{\mathit{\mathsf{i}}=\mathsf{1}}^{\mathit{\mathsf{N}}}{\displaystyle {\sum}_{\mathit{\mathsf{j}}=\mathsf{1}}^{\mathit{\mathsf{k}}}{\mathit{\mathsf{Y}}}_{\mathit{\mathsf{i}}\mathit{\mathsf{j}}}}}/\mathit{\mathsf{N}}$ and *N* is the total number of subject. Its variance can be obtained as

17 $$\mathit{\mathsf{V}}\mathit{\mathsf{a}}\mathit{\mathsf{r}}\left({\widehat{\delta}}_{\mathit{\mathsf{P}}\mathit{\mathsf{P}}}\right)=\frac{\mathsf{2}\left(\mathsf{1}-\rho \right){\sigma}^{\mathsf{2}}}{\mathit{\mathsf{k}}\mathit{\mathsf{N}}}=\frac{\mathsf{2}\left(\mathsf{1}/\rho -\mathsf{1}\right){\sigma}_{\mu}^{\mathsf{2}}}{\mathit{\mathsf{k}}\mathit{\mathsf{N}}}.$$

It is because from equations (12) and (13) we have

$$\begin{array}{c}\mathit{\mathsf{V}}\mathit{\mathsf{a}}\mathit{\mathsf{r}}\left({\overline{\mathit{\mathsf{S}}}}_{\mathit{\mathsf{p}\mathsf{ost}}}-{\overline{\mathit{\mathsf{S}}}}_{\mathit{\mathsf{p}}\mathit{\mathsf{r}}e}\right)=\mathit{\mathsf{V}}\mathit{\mathsf{a}}\mathit{\mathsf{r}}\left({\overline{\mathit{\mathsf{S}}}}_{\mathit{\mathsf{p}\mathsf{ost}}}\right)+\mathit{\mathsf{V}}\mathit{\mathsf{a}}\mathit{\mathsf{r}}\left({\overline{\mathit{\mathsf{S}}}}_{\mathit{\mathsf{p}}\mathit{\mathsf{r}}e}\right)-\mathsf{2}\mathit{\mathsf{C}}\mathit{\mathsf{o}}\mathit{\mathsf{v}}\left({\overline{\mathit{\mathsf{S}}}}_{\mathit{\mathsf{p}\mathsf{ost}}},{\overline{\mathit{\mathsf{S}}}}_{\mathit{\mathsf{p}}\mathit{\mathsf{r}}e}\right)\\ {}=\mathit{\mathsf{k}}{\sigma}^{\mathsf{2}}\left\{\mathsf{1}+\rho \left(\mathit{\mathsf{k}}-\mathsf{1}\right)\right\}/\mathit{\mathsf{N}}+\mathit{\mathsf{k}}{\sigma}^{\mathsf{2}}\left\{\mathsf{1}+\rho \left(\mathit{\mathsf{k}}-\mathsf{1}\right)\right\}/\mathit{\mathsf{N}}-\mathsf{2}{\mathit{\mathsf{k}}}^{\mathsf{2}}\rho {\sigma}^{\mathsf{2}}/\mathit{\mathsf{N}}\\ {}=\mathsf{2}\mathit{\mathsf{k}}{\sigma}^{\mathsf{2}}\left(\mathsf{1}-\rho \right)/\mathit{\mathsf{N}}=\mathsf{2}\mathit{\mathsf{k}}{\sigma}_{\mu}^{\mathsf{2}}\left(\mathsf{1}/\rho -\mathsf{1}\right)/\mathit{\mathsf{N}}\;.\end{array}$$

The following test statistic can then be used for testing H_0_: *δ* = 0

18 $${\mathit{\mathsf{T}}}_{\mathit{\mathsf{P}}\mathit{\mathsf{P}}}=\frac{{\widehat{\delta}}_{\mathit{\mathsf{P}}\mathit{\mathsf{P}}}}{\sqrt{\mathit{\mathsf{V}}\mathit{\mathsf{a}}\mathit{\mathsf{r}}\left({\widehat{\delta}}_{\mathit{\mathsf{P}}\mathit{\mathsf{P}}}\right)}}=\frac{\sqrt{\mathit{\mathsf{k}}\mathit{\mathsf{N}}}{\widehat{\delta}}_{\mathit{\mathsf{P}}\mathit{\mathsf{P}}}}{\sigma_{\mu}\sqrt{\mathsf{2}\left(\mathsf{1}/\rho -\mathsf{1}\right)}}=\frac{\sqrt{\mathit{\mathsf{N}}}\left({\overline{\mathit{\mathsf{S}}}}_{\mathit{\mathsf{p}\mathsf{ost}}}-{\overline{\mathit{\mathsf{S}}}}_{\mathit{\mathsf{p}}\mathit{\mathsf{r}}e}\right)}{\sigma_{\mu}\sqrt{\mathsf{2}\mathit{\mathsf{k}}\left(\mathsf{1}/\rho -\mathsf{1}\right)}}.$$

Now, the statistical power *φ*_*PP*_ of *T*_*PP*_ for detecting non-zero *δ*_*PP*_ can be expressed as follows:

19 $${\varphi}_{\mathit{\mathsf{P}}\mathit{\mathsf{P}}}=\varPhi \left\{\left|{\delta}_{\mathit{\mathsf{P}}\mathit{\mathsf{P}}}/{\sigma}_{\mu}\right|\sqrt{\frac{\mathit{\mathsf{k}}\mathit{\mathsf{N}}}{\mathsf{2}\left(\mathsf{1}/\rho -\mathsf{1}\right)}}-{\varPhi}^{-\mathsf{1}}\left(\mathsf{1}-\alpha /\mathsf{2}\right)\right\}.$$

This statistical power is an increasing function of *ρ* for a fixed *σ*_*μ*_, which we assume. It follows that the power is also an increasing function of *C*_*α*_ as seen next. When *δ*_*PP*_ is standardized by *σ*_*μ*_ and *ρ* is replaced by equation (7), equation (19) can further be expressed in terms of ${\varDelta}_{\mathit{\mathsf{P}}\mathit{\mathsf{P}}}={\delta}_{\mathit{\mathsf{P}}\mathit{\mathsf{P}}}/{\sigma}_{\mu }$ and *C*_*α*_ as follows:

20 $${\varphi}_{\mathit{\mathsf{P}}\mathit{\mathsf{P}}}=\varPhi \left\{\left|{\varDelta}_{\mathit{\mathsf{P}}\mathit{\mathsf{P}}}\right|\sqrt{\frac{\mathit{\mathsf{N}}}{\mathsf{2}\left(\mathsf{1}/{\mathit{\mathsf{C}}}_{\alpha }-\mathsf{1}\right)}}-{\varPhi}^{-\mathsf{1}}\left(\mathsf{1}-\alpha /\mathsf{2}\right)\right\}.$$

This power function is seen to be independent of *k*, the number of items. Stated differently, the power will be the same between two instruments with different numbers of items as long as their *C*_*α*_’s are the same even if the correlation of items will be smaller for the instrument with fewer items.

When sample size determination is needed for a study using an instrument of any number of items with a known *C*_*α*_ for a desired statistical power *φ*, typically 80 %, it can be determined from equation as follows:

21 $$\mathit{\mathsf{N}}=\frac{\mathsf{2}\left(\mathsf{1}/{\mathit{\mathsf{C}}}_{\alpha }-\mathsf{1}\right){\mathit{\mathsf{z}}}_{\alpha, \varphi}^{\mathsf{2}}}{\varDelta_{\mathit{\mathsf{P}}\mathit{\mathsf{P}}}^{\mathsf{2}}},$$

where

22 $${\mathit{\mathsf{z}}}_{\alpha, \varphi }={\varPhi}^{-\mathsf{1}}\left(\mathsf{1}-\alpha /\mathsf{2}\right)+{\varPhi}^{-\mathsf{1}}\left(\varphi \right).$$

The sample size (21) is seen to be a decreasing function of increasing *C*_*α*_ and Δ. In a possibly rare case in which determination of number of items with known correlations among them is needed for development of an instrument, it has to be determined from equation (19), instead of equation (20), as follow:

23 $$\mathit{\mathsf{k}}=\frac{\mathsf{2}\left(\mathsf{1}/\rho -\mathsf{1}\right){\mathit{\mathsf{z}}}_{\alpha, \varphi}^{\mathsf{2}}}{\mathit{\mathsf{N}}{\varDelta}_{\mathit{\mathsf{P}}\mathit{\mathsf{P}}}^{\mathsf{2}}}.$$

### Comparison of within-group effects between groups

In clinical trials, it is often of interest to compare within-group changes between groups. For instance, a clinical trial can be designed to compare of pre-post effect of an experimental treatment between treatment and control groups, that is, an interaction effect between group and time point. Based on model (2), the pre- and post-intervention item scores can be specified as ${\mathit{\mathsf{Y}}}_{\mathit{\mathsf{i}}\mathit{\mathsf{j}}}^{\mathit{\mathsf{p}}\mathit{\mathsf{r}}e\left(\mathsf{0}\right)}={\mu}_{\mathit{\mathsf{i}}}^{\left(\mathsf{0}\right)}+{e}_{\mathit{\mathsf{i}}\mathit{\mathsf{j}}}$ and ${\mathit{\mathsf{Y}}}_{\mathit{\mathsf{i}}\mathit{\mathsf{j}}}^{\mathit{\mathsf{post}}\left(\mathsf{0}\right)}={\mu}_{\mathit{\mathsf{i}}}^{\left(\mathsf{0}\right)}+{\delta}_{\mathsf{0}}+{e}_{\mathit{\mathsf{i}}\mathit{\mathsf{j}}}$ for the control group ${\mathit{\mathsf{Y}}}_{\mathit{\mathsf{i}}\mathit{\mathsf{j}}}^{\mathit{\mathsf{p}}\mathit{\mathsf{r}}e\left(\mathsf{1}\right)}={\mu}_{\mathit{\mathsf{i}}}^{\left(\mathsf{1}\right)}+{e}_{\mathit{\mathsf{i}}\mathit{\mathsf{j}}}$ and ${\mathit{\mathsf{Y}}}_{\mathit{\mathsf{i}}\mathit{\mathsf{j}}}^{\mathit{\mathsf{post}}\left(\mathsf{1}\right)}={\mu}_{\mathit{\mathsf{i}}}^{\left(\mathsf{1}\right)}+{\delta}_{\mathsf{1}}+{e}_{\mathit{\mathsf{i}}\mathit{\mathsf{j}}}$ for the treatment group. The primary interest will be testing H_o_: *δ*_*BW*_ = *δ*_1_ – *δ*_0_ = 0, i.e., whether or not the difference in pre-post differences between groups will be the same. Consequently, we have

24 $$E\left\{{D}_{trt}(S)\right\}-E\left\{{D}_{control}(S)\right\}=k{\delta}_{BW},$$

where ${\mathit{\mathsf{D}}}_{\mathit{\mathsf{t}}\mathit{\mathsf{r}}\mathit{\mathsf{t}}}\left(\mathit{\mathsf{S}}\right)={\mathit{\mathsf{S}}}_{\mathit{\mathsf{p}\mathsf{ost}}\left(\mathsf{1}\right)}-{\mathit{\mathsf{S}}}_{\mathit{\mathsf{p}}\mathit{\mathsf{r}}e\left(\mathsf{1}\right)}={\displaystyle {\sum}_{\mathit{\mathsf{j}}=\mathsf{1}}^{\mathit{\mathsf{k}}}{\mathit{\mathsf{Y}}}_{\mathit{\mathsf{i}}\mathit{\mathsf{j}}}^{\mathit{\mathsf{p}\mathsf{ost}}\left(\mathsf{1}\right)}}-{\displaystyle {\sum}_{\mathit{\mathsf{j}}=\mathsf{1}}^{\mathit{\mathsf{k}}}{\mathit{\mathsf{Y}}}_{\mathit{\mathsf{i}}\mathit{\mathsf{j}}}^{\mathit{\mathsf{p}}\mathit{\mathsf{r}}e\left(\mathsf{1}\right)}}$ and ${\mathit{\mathsf{D}}}_{\mathit{\mathsf{c}}\mathit{\mathsf{o}}n\mathit{\mathsf{trol}}}\left(\mathit{\mathsf{S}}\right)$ can be similarly defined. A moment estimate of *δ*_*BW*_ from (24) can be obtained as

25 $${\widehat{\delta}}_{\mathit{\mathsf{B}}\mathit{\mathsf{W}}}=\left({\overline{\mathit{\mathsf{D}}}}_{\mathit{\mathsf{t}}\mathit{\mathsf{r}}\mathit{\mathsf{t}}}-{\overline{\mathit{\mathsf{D}}}}_{\mathit{\mathsf{c}}\mathit{\mathsf{o}}n\mathit{\mathsf{t}\mathsf{rol}}}\right)/\mathit{\mathsf{k}},$$

where *N* is the number of subjects *per group*, $${\overline{\mathit{\mathsf{D}}}}_{\mathit{\mathsf{t}}\mathit{\mathsf{r}}\mathit{\mathsf{t}}}\equiv {\overline{\mathit{\mathsf{D}}}}_{\mathit{\mathsf{t}}\mathit{\mathsf{r}}\mathit{\mathsf{t}}}\left(\mathit{\mathsf{S}}\right)={\overline{\mathit{\mathsf{S}}}}_{\mathit{\mathsf{p}\mathsf{ost}}\left(\mathsf{1}\right)}-{\overline{\mathit{\mathsf{S}}}}_{\mathit{\mathsf{p}}\mathit{\mathsf{r}}e\left(\mathsf{1}\right)}=$$$${\displaystyle {\sum}_{\mathit{\mathsf{i}}=\mathsf{1}}^{\mathit{\mathsf{N}}}{\displaystyle {\sum}_{\mathit{\mathsf{j}}}^{\mathit{\mathsf{k}}}{\mathit{\mathsf{Y}}}_{\mathit{\mathsf{i}}\mathit{\mathsf{j}}}^{\mathit{\mathsf{p}\mathsf{ost}}\left(\mathsf{1}\right)}}}/\mathit{\mathsf{N}}-{\displaystyle {\sum}_{\mathit{\mathsf{i}}=\mathsf{1}}^{\mathit{\mathsf{N}}}{\displaystyle {\sum}_{\mathit{\mathsf{j}}}^{\mathit{\mathsf{k}}}{\mathit{\mathsf{Y}}}_{\mathit{\mathsf{i}}\mathit{\mathsf{j}}}^{\mathit{\mathsf{p}}\mathit{\mathsf{r}}e\left(\mathsf{1}\right)}}}/\mathit{\mathsf{N}}$$, and, $${\overline{\mathit{\mathsf{D}}}}_{\mathit{\mathsf{c}}\mathit{\mathsf{o}}n\mathit{\mathsf{trol}}}$$ can similarly be defined. The variance of $${\widehat{\delta}}_{\mathit{\mathsf{W}}\mathit{\mathsf{B}}}$$ is

26 $$\mathit{\mathsf{V}}\mathit{\mathsf{a}}\mathit{\mathsf{r}}\left({\widehat{\delta}}_{\mathit{\mathsf{B}}\mathit{\mathsf{W}}}\right)=\frac{\mathsf{4}\left(\mathsf{1}-\rho \right){\sigma}^{\mathsf{2}}}{\mathit{\mathsf{k}}\mathit{\mathsf{N}}}=\frac{\mathsf{4}\left(\mathsf{1}/\rho -\mathsf{1}\right){\sigma}_{\mu}^{\mathsf{2}}}{\mathit{\mathsf{k}}\mathit{\mathsf{N}}}.$$

Therefore, the following test statistic can be used for testing the null hypothesis H_o_: *δ*_*BW*_ = 0,

27 $${\mathit{\mathsf{T}}}_{\mathit{\mathsf{B}}\mathit{\mathsf{W}}}=\frac{{\widehat{\delta}}_{\mathit{\mathsf{B}}\mathit{\mathsf{W}}}}{\sqrt{\mathit{\mathsf{V}}\mathit{\mathsf{a}}\mathit{\mathsf{r}}\left({\widehat{\delta}}_{\mathit{\mathsf{B}}\mathit{\mathsf{W}}}\right)}}=\frac{\sqrt{\mathit{\mathsf{k}}\mathit{\mathsf{N}}}{\widehat{\delta}}_{\mathit{\mathsf{B}}\mathit{\mathsf{W}}}}{\mathsf{2}{\sigma}_{\mu}\sqrt{\left(\mathsf{1}/\rho -\mathsf{1}\right)}}=\frac{\sqrt{\mathit{\mathsf{N}}}\left({\overline{\mathit{\mathsf{D}}}}_{\mathit{\mathsf{t}}\mathit{\mathsf{r}}\mathit{\mathsf{t}}}-{\overline{\mathit{\mathsf{D}}}}_{\mathit{\mathsf{c}}\mathit{\mathsf{o}}n\mathit{\mathsf{t}\mathsf{rol}}}\right)}{\mathsf{2}{\sigma}_{\mu}\sqrt{\mathit{\mathsf{k}}\left(\mathsf{1}/\rho -\mathsf{1}\right)}}.$$

The statistical power *φ*_*BW*_ of *T*_*BW*_ for detecting non-zero *δ*_*BW*_ can thus be expressed as follows:

28 $${\varphi}_{\mathit{\mathsf{B}}\mathit{\mathsf{W}}}=\varPhi \left\{\left|{\delta}_{\mathit{\mathsf{B}}\mathit{\mathsf{W}}}/{\sigma}_{\mu}\right|\sqrt{\frac{\mathit{\mathsf{k}}\mathit{\mathsf{N}}}{\mathsf{4}\left(\mathsf{1}/\rho -\mathsf{1}\right)}}-{\varPhi}^{-\mathsf{1}}\left(\mathsf{1}-\alpha /\mathsf{2}\right)\right\}.$$

Again, this statistical power is an increasing of *ρ* and of *C*_*α*_ as well as seen next. When *δ*_*BW*_ is standardized by *σ*_*μ*_ and *ρ* is replaced by equation (7), equation (28) can further be expressed in terms of ${\varDelta}_{\mathit{\mathsf{B}}\mathit{\mathsf{W}}}={\delta}_{\mathit{\mathsf{B}}\mathit{\mathsf{W}}}/{\sigma}_{\mu }$ and *C*_*α*_ as follows:

29 $${\varphi}_{\mathit{\mathsf{B}}\mathit{\mathsf{W}}}=\varPhi \left\{\left|{\varDelta}_{\mathit{\mathsf{B}}\mathit{\mathsf{W}}}\right|\sqrt{\frac{\mathit{\mathsf{N}}}{\mathsf{4}\left(\mathsf{1}/{\mathit{\mathsf{C}}}_{\alpha }-\mathsf{1}\right)}}-{\varPhi}^{-\mathsf{1}}\left(\mathsf{1}-\alpha /\mathsf{2}\right)\right\}.$$

Again, this power function is seen to be independent of *k*, the number of items.

Sample size for a desired statistical power *φ* can be determined from (27) as follows:

30 $$\mathit{\mathsf{N}}=\frac{\mathsf{4}\left(\mathsf{1}/{\mathit{\mathsf{C}}}_{\alpha }-\mathsf{1}\right){\mathit{\mathsf{z}}}_{\alpha, \varphi}^{\mathsf{2}}}{\varDelta_{\mathit{\mathsf{B}}\mathit{\mathsf{W}}}^{\mathsf{2}}}.$$

Again, this sample size (30) is seen to be a decreasing function of increasing *C*_*α*_ and Δ. When number of items is needed for development of an instrument, it can be determined from equation (28) as follow:

31 $$\mathit{\mathsf{k}}=\frac{\mathsf{2}\left(\mathsf{1}/\rho -\mathsf{1}\right){\mathit{\mathsf{z}}}_{\alpha, \varphi}^{\mathsf{2}}}{\mathit{\mathsf{N}}{\varDelta}_{\mathit{\mathsf{B}}\mathit{\mathsf{W}}}^{\mathsf{2}}}.$$

### Two-sample between-group comparison

Comparison of means between groups using an instrument is widely tested in clinical trials. Based on model (2), the intervention item scores from control and treatment groups can be specified as ${\mathit{\mathsf{Y}}}_{\mathit{\mathsf{i}}\mathit{\mathsf{j}}}^{\left(\mathsf{0}\right)}={\mu}_{\mathit{\mathsf{i}}}+{e}_{\mathit{\mathsf{i}}\mathit{\mathsf{j}}}$ and ${\mathit{\mathsf{Y}}}_{\mathit{\mathsf{i}}\mathit{\mathsf{j}}}^{\left(\mathsf{1}\right)}={\mu}_{\mathit{\mathsf{i}}}+{\delta}_{\mathit{\mathsf{T}}\mathit{\mathsf{S}}}+{e}_{\mathit{\mathsf{i}}\mathit{\mathsf{j}}}$, respectively. The primary interest will be testing H_o_: *δ*_*TS*_ = 0, i.e., whether or not the means are the same between the two groups. Under this formulation, we have

32 $$E\left({S}_{trt}\right)=E\left({S}_{control}\right)+k{\delta}_{TS},$$

where ${\mathit{\mathsf{S}}}_{\mathit{\mathsf{t}}\mathit{\mathsf{r}}\mathit{\mathsf{t}}}={\displaystyle {\sum}_{\mathit{\mathsf{j}}=\mathsf{1}}^{\mathit{\mathsf{k}}}{\mathit{\mathsf{Y}}}_{\mathit{\mathsf{i}}\mathit{\mathsf{j}}}^{\left(\mathsf{1}\right)}}$ and ${\mathit{\mathsf{S}}}_{\mathit{\mathsf{c}}\mathit{\mathsf{o}}n\mathit{\mathsf{trol}}}={\displaystyle {\sum}_{\mathit{\mathsf{j}}=\mathsf{1}}^{\mathit{\mathsf{k}}}{\mathit{\mathsf{Y}}}_{\mathit{\mathsf{i}}\mathit{\mathsf{j}}}^{\left(\mathsf{0}\right)}}$ represents scale scores under treatment and control groups, respectively. A moment estimate of *δ*_*TS*_ can be obtained from (32) as

33 $${\widehat{\delta}}_{\mathit{\mathsf{T}}\mathit{\mathsf{S}}}=\left({\overline{\mathit{\mathsf{S}}}}_{\mathit{\mathsf{t}}\mathit{\mathsf{r}}\mathit{\mathsf{t}}}-{\overline{\mathit{\mathsf{S}}}}_{\mathit{\mathsf{c}}\mathit{\mathsf{o}}n\mathit{\mathsf{t}\mathsf{rol}}}\right)/\mathit{\mathsf{k}},$$

where ${\overline{\mathit{\mathsf{S}}}}_{\mathit{\mathsf{t}}\mathit{\mathsf{r}}\mathit{\mathsf{t}}}={\displaystyle {\sum}_{\mathit{\mathsf{i}}=\mathsf{1}}^{\mathit{\mathsf{N}}}{\displaystyle {\sum}_{\mathit{\mathsf{j}}=\mathsf{1}}^{\mathit{\mathsf{k}}}{\mathit{\mathsf{Y}}}_{\mathit{\mathsf{i}}\mathit{\mathsf{j}}}^{\left(\mathsf{1}\right)}}}/\mathit{\mathsf{N}}$, ${\overline{\mathit{\mathsf{S}}}}_{\mathit{\mathsf{c}}\mathit{\mathsf{o}}n\mathit{\mathsf{trol}}}={\displaystyle {\sum}_{\mathit{\mathsf{i}}=\mathsf{1}}^{\mathit{\mathsf{N}}}{\displaystyle {\sum}_{\mathit{\mathsf{j}}=\mathsf{1}}^{\mathit{\mathsf{k}}}{\mathit{\mathsf{Y}}}_{\mathit{\mathsf{i}}\mathit{\mathsf{j}}}^{\left(\mathsf{0}\right)}}}/\mathit{\mathsf{N}}$ and *N* is the number of participants per group. The variance of ${\widehat{\delta}}_{\mathit{\mathsf{T}}\mathit{\mathsf{S}}}$ can be obtained as

34 $$\mathit{\mathsf{V}}\mathit{\mathsf{a}}\mathit{\mathsf{r}}\left({\widehat{\delta}}_{\mathit{\mathsf{T}}\mathit{\mathsf{S}}}\right)=\frac{\mathsf{2}\left\{\mathsf{1}+\rho \left(\mathit{\mathsf{k}}-\mathsf{1}\right)\right\}{\sigma}^{\mathsf{2}}}{\mathit{\mathsf{k}}\mathit{\mathsf{N}}}=\frac{\mathsf{2}\left\{\mathsf{1}/\rho +\mathit{\mathsf{k}}-\mathsf{1}\right\}{\sigma}_{\mu}^{\mathsf{2}}}{\mathit{\mathsf{k}}\mathit{\mathsf{N}}}.$$

The corresponding test statistic *T*_*TS*_ can be built as

35 $${\mathit{\mathsf{T}}}_{\mathit{\mathsf{T}}\mathit{\mathsf{S}}}=\frac{{\widehat{\delta}}_{\mathit{\mathsf{T}}\mathit{\mathsf{S}}}}{\sqrt{\mathit{\mathsf{V}}\mathit{\mathsf{a}}\mathit{\mathsf{r}}\left({\widehat{\delta}}_{\mathit{\mathsf{T}}\mathit{\mathsf{S}}}\right)}}=\frac{\sqrt{\mathit{\mathsf{k}}\mathit{\mathsf{N}}}{\widehat{\delta}}_{\mathit{\mathsf{T}}\mathit{\mathsf{S}}}}{\sigma_{\mu}\sqrt{\mathsf{2}\left(\mathsf{1}/\rho +\mathit{\mathsf{k}}-\mathsf{1}\right)}}=\frac{\sqrt{\mathit{\mathsf{N}}}\left({\overline{\mathit{\mathsf{S}}}}_{\mathit{\mathsf{t}}\mathit{\mathsf{r}}\mathit{\mathsf{t}}}-{\overline{\mathit{\mathsf{S}}}}_{\mathit{\mathsf{c}}\mathit{\mathsf{o}}n\mathit{\mathsf{t}\mathsf{rol}}}\right)}{\sigma_{\mu}\sqrt{\mathsf{2}\mathit{\mathsf{k}}\left(\mathsf{1}/\rho +\mathit{\mathsf{k}}-\mathsf{1}\right)}}.$$

And the power function *φ*_*TS*_ of *T*_*TS*_ can be expressed as

36 $${\varphi}_{\mathit{\mathsf{T}}\mathit{\mathsf{S}}}=\varPhi \left\{\left|{\delta}_{\mathit{\mathsf{T}}\mathit{\mathsf{S}}}/{\sigma}_{\mu}\right|\sqrt{\frac{\mathit{\mathsf{k}}\mathit{\mathsf{N}}}{\mathsf{2}\left(\mathsf{1}/\rho +\mathit{\mathsf{k}}-\mathsf{1}\right)}}-{\varPhi}^{-\mathsf{1}}\left(\mathsf{1}-\alpha /\mathsf{2}\right)\right\}.$$

It should be noted that this statistical power (36) is also an increasing function of *ρ* in contrast to a situation when a fixed total variance assumption is more reasonable in which both ${\sigma}_e^{\mathsf{2}}$ and ${\sigma}_{\mu}^{\mathsf{2}}$ are a function of *ρ* but σ^2^ is not. For example, observations without measurement errors from clusters are often assumed to be correlated and power of between-group tests using such correlated observations is a decreasing function of *ρ* [18]. Again, when *δ*_*TS*_ is standardized by *σ*_*μ*_ and *ρ* is replaced by equation (7), equation (33) can further be expressed in terms can further be expressed in terms of ${\varDelta}_{\mathit{\mathsf{T}}\mathit{\mathsf{S}}}={\delta}_{\mathit{\mathsf{T}}\mathit{\mathsf{S}}}/{\sigma}_{\mu }$ and *C*_*α*_ as follows:

37 $${\varphi}_{\mathit{\mathsf{T}}\mathit{\mathsf{S}}}=\varPhi \left\{\left|{\varDelta}_{\mathit{\mathsf{T}}\mathit{\mathsf{S}}}\right|\sqrt{{\mathit{\mathsf{C}}}_{\alpha}\mathit{\mathsf{N}}/\mathsf{2}}-{\varPhi}^{-\mathsf{1}}\left(\mathsf{1}-\alpha /\mathsf{2}\right)\right\}.$$

Again, this power function is seen to be independent of *k*, the number of items.

Sample size for a desired statistical power *φ* can be determined from (37) as follows:

38 $$\mathit{\mathsf{N}}=\frac{\mathsf{2}{\mathit{\mathsf{z}}}_{\alpha, \varphi}^{\mathsf{2}}}{{\mathit{\mathsf{C}}}_{\alpha }{\varDelta}_{\mathit{\mathsf{B}}\mathit{\mathsf{W}}}^{\mathsf{2}}}.$$

Again, the sample size (38) is seen to be a decreasing function of increasing *C*_*α*_ and Δ. When number of items is needed for development of an instrument, it can be determined from equation (36) as follow:

39 $$\mathit{\mathsf{k}}=\frac{\mathsf{2}\left(\mathsf{1}/\rho -\mathsf{1}\right){\mathit{\mathsf{z}}}_{\alpha, \varphi}^{\mathsf{2}}/{\varDelta}_{\mathit{\mathsf{T}}\mathit{\mathsf{S}}}^{\mathsf{2}}}{\mathit{\mathsf{N}}-\mathsf{2}{\mathit{\mathsf{z}}}_{\alpha, \varphi}^{\mathsf{2}}/{\varDelta}_{\mathit{\mathsf{T}}\mathit{\mathsf{S}}}^{\mathsf{2}}}.$$

## Results

To validate equation (14) and the power functions (20), (29), and (37), we conduct simulation study for each test. For the simulation, the random item scores are generated based on model (2) assuming both *μ*_*i*_ and *e*_*ij*_ are normally distributed although this assumption is not required in general. Under this normal assumption, however, it can be shown that all the moment estimates herein are the maximum likelihood estimates [19]. We then compute scale scores by summing up the item scores for each individual.

We fix a two-tailed significance level of *α* = 0.05 and ${\sigma}_{\mu}^{\mathsf{2}}$ = 1 without loss generality for all simulations, and determine ${\sigma}_e^{\mathsf{2}}$ and σ^2^ through *ρ* determined by given *k* and *C*_*α*_. We randomly generate 1000 data sets for each combination of design parameters that include effect size Δ, number of items *k*, and sample size *N*. We then compute empirical power $\tilde{\varphi}$ by counting data sets from which two-tailed p-values are smaller than 0.05; that is, $\tilde{\varphi}={\displaystyle {\sum}_{\mathit{\mathsf{s}}}^{\mathsf{1000}}\mathsf{1}\left({\mathit{\mathsf{p}}}_{\mathit{\mathsf{s}}}<\alpha \right)}/\mathsf{1000}$ where *p*_*s*_ represents a two-sided p-value from the *s*-th simulated data set. For the testing, we applied corresponding t-tests assuming the variances of the moment estimates are unknown, which is practically reasonable. We used SAS v9.3 for the simulations.

### Test-retest correlation

The results are presented in Table 1 that shows the empirically estimated test-retest correlations (i.e., average of 1000 estimated Pearson correlations for each set of design parameter specifications) are approximately the same as the pre-assigned *C*_*α*_, regardless of sample size *N*, which is as small as 30, and number of items *k*. Therefore, equality between *C*_*α*_ and test-retest correlation (14) is well validated.

**Table 1:** Empirical simulation-based estimates of test-retest correlation *Corr*(*S*_*test*_, *S*_*retest*_) in equation (14)

	*Corr*(*S*_*test*_, *S*_*retest*_)			
	Total *N* = 30		Total *N* = 50	
*C*_*α*_	*k* = 5	*k* = 10	*k* = 5	*k* = 10
0.1	0.10	0.10	0.10	0.10
0.2	0.20	0.20	0.20	0.20
0.3	0.30	0.29	0.30	0.30
0.4	0.39	0.39	0.40	0.39
0.5	0.49	0.50	0.49	0.50
0.6	0.59	0.59	0.60	0.60
0.7	0.69	0.69	0.70	0.70
0.8	0.79	0.80	0.80	0.79
0.9	0.90	0.90	0.90	0.90

Note: Total *N*: total number of subjects; *C*_*α*_: Cronbach alpha; *k*: number of items

### Pre-post intervention comparison

Table 2 shows that the theoretical power *φ*_*PP*_ (20) is very close to the empirical power ${\tilde{\varphi}}_{\mathit{\mathsf{P}}\mathit{\mathsf{P}}}$ obtained through the simulations. The results validate that the power *φ*_*PP*_ increases with increasing *C*_*α*_ (or equivalently increasing correlation for the same *k*) in the “pre-post” test settings, regardless of sample size *N* and number of items *k*. Furthermore, it shows that the statistical power does not depend on *k* for a given *C*_*α*_ even if correlation *ρ* does.

**Table 2:** Statistical power of the pre-post test *T*_*PP*_ (18): *σ*_*μ*_ = 1

			*k* = 5		*k* = 10	
Total *N*	*Δ*_*PP*_	*C*_*α*_	*φ*_*PP*_	${\tilde{\varphi}}_{\mathit{\mathsf{P}}\mathit{\mathsf{P}}}$	*φ*_*PP*_	${\tilde{\varphi}}_{\mathit{\mathsf{P}}\mathit{\mathsf{P}}}$
30	0.4	0.5	0.341	0.337	0.341	0.310
		0.6	0.475	0.459	0.475	0.458
		0.7	0.658	0.626	0.658	0.649
		0.8	0.873	0.849	0.873	0.830
		0.9	0.996	0.997	0.996	0.995
50	0.3	0.5	0.323	0.309	0.323	0.296
		0.6	0.451	0.424	0.451	0.433
		0.7	0.630	0.633	0.630	0.614
		0.8	0.851	0.849	0.851	0.844
		0.9	0.994	0.995	0.994	0.992

Note: Total *N*: total number of subjects; *k*: number of items; ${\varDelta}_{\mathit{\mathsf{P}}\mathit{\mathsf{P}}}={\delta}_{\mathit{\mathsf{P}}\mathit{\mathsf{P}}}/{\sigma}_{\mu }$; *C*_*α*_: Cronbach alpha; *φ*_*PP*_: theoretical power (20); ${\tilde{\varphi}}_{\mathit{\mathsf{P}}\mathit{\mathsf{P}}}$: simulation-based empirical power

### Between-group whithin-group comparison

Table 3 shows that the theoretical power *φ*_*BW*_ (29) is very close to the empirical power ${\tilde{\varphi}}_{\mathit{\mathsf{B}}\mathit{\mathsf{W}}}$ obtained through the simulations. Therefore, the results validate that the statistical power *φ*_*BW*_ increases with increasing *C*_*α*_ for testing hypotheses concerning between-group effects on within-group changes regardless of *N*, sample size per group, and *k*. Again, it shows that the statistical power does not depend on *k* for a given *C*_*α*_ even if correlation *ρ* does.

**Table 3:** Statistical power of the between-group within-group test *T*_*BW*_ (25): *σ*_*μ*_ = 1

			*k* = 5		*k* = 10	
*N* per group	*Δ*_*BW*_	*C*_*α*_	*φ*_*BW*_	${\tilde{\varphi}}_{\mathit{\mathsf{B}}\mathit{\mathsf{W}}}$	*φ*_*BW*_	${\tilde{\varphi}}_{\mathit{\mathsf{B}}\mathit{\mathsf{W}}}$
30	0.4	0.5	0.194	0.179	0.183	0.194
		0.6	0.268	0.264	0.254	0.268
		0.7	0.387	0.375	0.359	0.387
		0.8	0.591	0.618	0.594	0.591
		0.9	0.908	0.884	0.901	0.908
50	0.3	0.5	0.164	0.184	0.214	0.184
		0.6	0.242	0.254	0.261	0.254
		0.7	0.387	0.367	0.365	0.367
		0.8	0.511	0.564	0.591	0.564
		0.9	0.893	0.889	0.893	0.889

Note: *N* per group: number of subjects per group; *k*: number of items; ${\varDelta}_{\mathit{\mathsf{B}}\mathit{\mathsf{W}}}={\delta}_{\mathit{\mathsf{B}}\mathit{\mathsf{W}}}/{\sigma}_{\mu }$; *C*_*α*_: Cronbach alpha; *φ*_*BW*_: theoretical power (27); ${\tilde{\varphi}}_{\mathit{\mathsf{B}}\mathit{\mathsf{W}}}$: simulation-based empirical power

### Two-sample between-group comparison

Table 4 shows again that the theoretical power *φ*_*TS*_ (37) is very close to the empirical power ${\tilde{\varphi}}_{\mathit{\mathsf{T}}\mathit{\mathsf{S}}}$ obtained through the simulations. The results validate that the statistical power increases with increasing Cronbach *α* even for two-sample testing in cross-sectional settings that does not involve within-group effects. it shows that the statistical power does not depend on *k* for a given *C*_*α*_ even if correlation *ρ* does. Again, it shows that the statistical power does not depend on *k* for a given *C*_*α*_ even if correlation *ρ* does.

**Table 4:** Statistical power of the between-group within-group test *T*_*TS*_ (32): *σ*_*μ*_ = 1

			*k* = 5		*k* = 10	
*N* per group	*Δ*_*TS*_	*C*_*α*_	*φ*_*TS*_	${\tilde{\varphi}}_{\mathit{\mathsf{T}}\mathit{\mathsf{S}}}$	*φ*_*TS*_	${\tilde{\varphi}}_{\mathit{\mathsf{T}}\mathit{\mathsf{S}}}$
50	0.7	0.5	0.697	0.676	0.697	0.697
		0.6	0.774	0.758	0.774	0.760
		0.7	0.834	0.812	0.834	0.813
		0.8	0.879	0.872	0.879	0.882
		0.9	0.913	0.901	0.913	0.895
100	0.5	0.5	0.705	0.682	0.705	0.679
		0.6	0.782	0.791	0.782	0.769
		0.7	0.841	0.820	0.841	0.832
		0.8	0.885	0.879	0.885	0.908
		0.9	0.918	0.929	0.918	0.912

Note: *N* per group: number of subjects per group; *k*: number of items; ${\varDelta}_{\mathit{\mathsf{T}}\mathit{\mathsf{S}}}={\delta}_{\mathit{\mathsf{T}}\mathit{\mathsf{S}}}/{\sigma}_{\mu }$; *C*_*α*_: Cronbach alpha; *φ*_*TS*_: theoretical power (34); ${\tilde{\varphi}}_{\mathit{\mathsf{T}}\mathit{\mathsf{S}}}$: simulation-based empirical power

## Discussion

We demonstrate by deriving explicit power functions that higher internal consistency or reliability of unidimensional parallel instrument items measured by Cronbach alpha *C*_*α*_ results in greater statistical power of several tests regardless of whether comparisons are made within or between groups. In addition, the test-retest reliability correlation of such items is shown to be the same as Cronbach alpha *C*_*α*_. Due to this property, testing significance of *C*_*α*_ can be equivalent to testing that of a correlation through the Fisher z-transformation. Furthermore, all of the power functions derived herein can even be applied to trials using single item instrument with measurement error since the power function depends only on *C*_*α*_ which can be estimated via test-retest correlations for single item instruments as mentioned earlier. The demonstrations are made theoretically, and validations are made through simulation studies that show that the derived test statistics and their corresponding power functions are very close to each other. Therefore, the sample size determination formulas (21), (30), and (38) are valid and so are the determinations of number of items (22), (31), and (39) in different settings.

In fact, for longitudinal studies aiming to compare within-group effects using such as *T*_*PP*_ (18) and *T*_*BW*_ (27), the fixed true score variance assumption is not critical since the true score *μ*_*i*_’s in model (2) are cancelled by taking differences of *Y* between pre and post-interventions and thus makes the variance of the pre-post differences depend only on measurement error variance ${\sigma}_e^{\mathsf{2}}$. For example, the variance equations (17) and (26) can be expressed in term of only ${\sigma}_e^{\mathsf{2}}$, a decreasing function of *ρ*, through equation (4) as follows: $\mathit{\mathsf{V}}\mathit{\mathsf{a}}\mathit{\mathsf{r}}\left({\widehat{\delta}}_{\mathit{\mathsf{P}}\mathit{\mathsf{P}}}\right)=\mathsf{2}{\sigma}_e^{\mathsf{2}}/\left(\mathit{\mathsf{k}}\mathit{\mathsf{N}}\right)$ and $\mathit{\mathsf{V}}\mathit{\mathsf{a}}\mathit{\mathsf{r}}\left({\widehat{\delta}}_{\mathit{\mathsf{B}}\mathit{\mathsf{W}}}\right)=\mathsf{4}{\sigma}_e^{\mathsf{2}}/\left(\mathit{\mathsf{k}}\mathit{\mathsf{N}}\right)$. In other words, both the power functions *φ*_*PP*_ (20) and *φ*_*BW*_ (29) are increasing function of *C*_*α*_ or *ρ* regardless of whether total variance or true score variance is assumed fixed.

In contrast, however, for cross-sectional studies aiming to compare between-group effects using *T*_*TS*_ (35), the fixed true score variance assumption is critical since the variance equation (34) cannot be expressed only in term of only ${\sigma}_e^{\mathsf{2}}$, and furthermore it can be shown that under a fixed total variance assumption $\mathit{\mathsf{V}}\mathit{\mathsf{a}}\mathit{\mathsf{r}}\left({\widehat{\delta}}_{\mathit{\mathsf{T}}\mathit{\mathsf{S}}}\right)$ (34) is an increasing function of *ρ* (see equation (10)) and so is the power function. In sum, the fixed true score variance assumption enables all of the power functions to be an increasing function of *C*_*α*_ or *ρ* in a unified fashion. For example, Leon *et al.* [20] used a real data set of HRSD ratings to empirically demonstrate that the statistical power of a two-sample between-group test is increasing with increased *C*_*α*_, although they increased *C*_*α*_ by increasing number of items *k*, not necessarily by increasing *ρ* for a fixed number of items.

In most cases, item scores are designed to be binary or ordinal scores on a likert scale. Therefore, the applicability of the derived power functions and sample size formulas to such cases could be in question since the scores are not normally distributed. Furthermore, it is not easy to build a model like (2) for non-normal scores particularly because measurement error variances depend on the true construct value. For example, variance of a binary score is a function of its mean. Perhaps, construction of marginal models in the sense of generalized estimating equations [21] can be considered for derivation of power functions assumption even if this approach is beyond the scope of the present study. After all, we believe that our study results should be able to be applied to non-normal scores by virtue of the central limit theorem. Another prominent limitation of our study is the very strong assumption of essentially τ-equivalent parallel items which may not be realistic at all [8], albeit conceivable for a unidimensional construct. Therefore, further development of power functions under relaxed conditions reflecting more real world situations should be a valuable future study.

## Conclusion

Instruments with greater Cronbach alpha should be used for any type of research since they have smaller measurement error and thus have greater statistical power for any research settings, cross-sectional or longitudinal. However, when items are parallel targeting a unidimensional construct, Cronbach alpha of an instrument should be enhanced by developing a set of highly correlated items but not by unduly increasing the number of items with inadequate inter-item correlations.

## Abbreviations

HRSD: Hamilton Rating Scale of Depression
